# Visual Aids for Multimodal Treatment Options to Support Decision Making of Patients with Colorectal Cancer

**DOI:** 10.1186/1472-6947-12-118

**Published:** 2012-10-23

**Authors:** Sabine Hofmann, Janina Vetter, Christiane Wachter, Doris Henne-Bruns, Franz Porzsolt, Marko Kornmann

**Affiliations:** 1 Department of General and Visceral Surgery, University of Ulm, Albert-Einstein-Allee 23, 89081, Ulm, Germany; 2 Study Group of Health Services Research at the Department of General and Visceral Surgery, University of Ulm, Albert-Einstein-Allee 23, 89081, Ulm, Germany

**Keywords:** Visual aids, Colorectal cancer, Quality of life, Side effects, Shared decision-making

## Abstract

**Background:**

A variety of multimodal treatment options are available for colorectal cancer and many patients want to be involved in decisions about their therapies. However, their desire for autonomy is limited by lack of disease-specific knowledge. Visual aids may be helpful tools to present complex data in an easy-to-understand, graphic form to lay persons. The aim of the present study was to evaluate the treatment preferences of healthy persons and patients using visual aids depicting multimodal treatment options for colorectal cancer.

**Methods:**

We designed visual aids for treatment scenarios based on four key studies concerning multimodal treatment of colorectal cancer. The visual aids were composed of diagrams depicting outcome parameters and side effects of two treatment options. They were presented to healthy persons (n = 265) and to patients with colorectal cancer (n = 102).

**Results:**

Most patients and healthy persons could make immediate decisions after seeing the diagrams (range: 88% – 100%). Patients (79%) chose the intensive-treatment option in the scenario with a clear survival benefit. In scenarios without survival benefit, all groups clearly preferred the milder treatment option (range: 78% - 90%). No preference was seen in the scenario depicting equally intense treatment options with different timing (neoadjuvant vs. adjuvant) but without survival benefit.

**Conclusions:**

Healthy persons’ and patients’ decisions using visual aids seem to be influenced by quality-of-life aspects rather than recurrence rates especially in situations without survival benefit. In the future visual aids may help to improve the management of patients with colorectal cancer.

## Background

Colorectal cancer (CRC) is the second most common form of cancer in both genders today. The last decades have shown a rising incidence, while advances in treatment have led to decreasing mortality [1]. Multimodal treatment strategies have been established and are presently regarded as standard for many tumor stages. The wealth of possible treatment options requires a sophisticated planning of the therapeutic approach. Recommendations for the individual treatment of patients are nowadays often made by an interdisciplinary panel of specialists based on the staging and pathological results.

However, the relation between the doctor and patients has changed considerably in recent years due to patients’ increasing desire for autonomy. Many patients want to be involved in the process of decision-making about their therapies [2] and already have differentiated ideas about their therapy stemming from information often gathered in the internet [3]. However, these ideas may be unrealistic because patients lack sufficient disease-specific knowledge and experience with the potential progression of the disease, whereas physicians may be misled in regard to patients’ expectations. Both patients and physicians are forced to make vital decisions in the face of complex and sometimes confusing data with regard to effectiveness and toxicity of various potential treatment options.

To be able to participate in decision-making it is essential to provide patients with information based on current scientific evidence that is both easy to understand and detailed. In malignant diseases such as breast or prostate cancer decision aids have been tested to facilitate the patient’s decision-making process [4-6]. In colorectal cancer decision aids were used in the process of screening [7,8]. Only one study so far evaluated decision aids for palliative colorectal cancer treatment [9]. Therefore, it is presently completely unknown how healthy persons and patients would decide when confronted with multimodal treatment options in curative settings of colorectal cancer. In contrast to decision aids which by definition have to be publicly available we recently designed visual aids for several scenarios of multimodal potentially curative treatment options of colorectal cancer which could be used by the physician to present the outcome and side effects of various treatment options to the patient [10,11]. The aim of the present study was to evaluate the treatment preferences of healthy persons and patients for potentially curative multimodal treatment options in colorectal cancer using these visual aids.

## Methods

### Selection of studies for visual aids

Four key studies of colorectal cancer influencing treatment strategies were chosen as a basis for the visual aid scenarios [12-19]. All studies were prospective, randomized controlled trials (Table 1). In scenarios 1 – 3, a mild therapeutic option consisting of an operation alone or an operation with adjuvant mono-chemotherapy was presented in comparison with a more intensive therapeutic option consisting of an operation with mono-chemotherapy, double-chemotherapy, or radiation [12,14,15]. In scenario 4, the results of two equally intense options were presented comparing neoadjuvant chemoradiotherapy with adjuvant chemoradiotherapy in rectal cancer [19].

**Table 1:** Study characteristics

**Scenario**	**Reference**	**Disease**	**UICC Stage**	**Mild Therapy**	**Intense Therapy**	**Characteristics**
**1**	[12,13]	colon cancer	III	OP only	OP + adjuvant 5-FU	survival benefit of intensive therapy
**2**	[14]	colon cancer	II + III	adjuvant 5 FU	adjuvant 5 FU + Oxaliplatin	no survival benefit
**3**	[15-18]	rectal cancer	I – III	OP only	OP + short-course radiotherapy	no survival benefit
	**Reference**	**Disease**	**UICC Stage**	**Pre-OP Option**	**Post-OP Option**	**Characteristics**
**4**	[19]	rectal cancer	II + III	preoperative CRT	postoperative CRT	no survival benefit

OP, operation; CRT, chemoradiotherapy.

### Design of the visual aids

**Figure 1:**
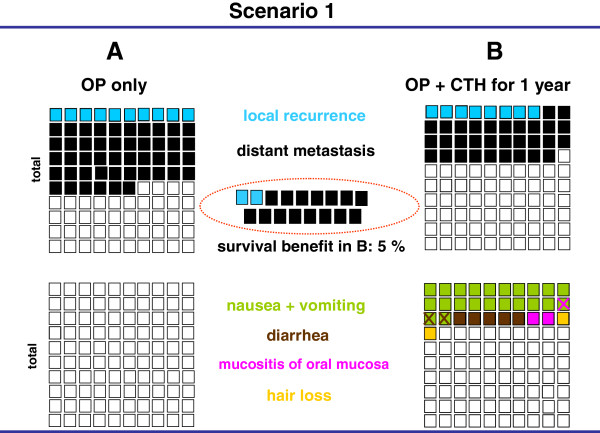
**Visual aid illustrating two treatment scenarios for patients with UICC stage III colon cancer based on the published data**[12,13]**.** The results of an operation (OP) alone (**A**) are shown as mild treatment on the left, and the results of an OP plus adjuvant 5-FU chemotherapy (CHT) (**B**) are shown as intense treatment on the right. The illustrations are based on 4 quadrants showing 100 squares representing% or events. The relative incidence of local recurrence and distant metastasis are displayed in the two upper quadrants and important undesired events in the two lower quadrants. The difference in recurrence of the two treatment options is summarized in the central dotted circle followed by a statement about overall survival benefit. To better differentiate the criteria, each is represented in a different color. If several criteria applied to one patient (e.g., nausea and diarrhea), the first criterion is shown as background color. Further criteria are shown in the foreground with the sign (X) in the appropriate color.

There exists a huge literature about how to present information to healthy persons and patients [20-22]. One of the key findings was that graphical presentation including frequencies may be most appropriate to present results of clinical trials [23]. Our designed visual aids depicted separately the results of two treatment options evaluated in a randomized controlled trial according to therapeutic outcome and side-effects. They were presented as graphs as described previously [10,11]. This was achieved by four-colored square diagrams, each consisting of 100 small squares (four quadrants), representing a total of 100 patients or 100% (Figure 1). One colored square stands for one event in a patient; a cross means the coincidence of two events in one patient. The two quadrants on the left side show the first treatment option, while the second option is displayed in the quadrants on the right side. The two upper quadrants show the outcome results, such as mortality, local recurrence rates and distant metastases. The two lower quadrants display possible side effects. Survival differences between the two therapy arms are presented separately in the middle (Figure 1).

Out of the recommended quality criteria for patient decision aids published [20,24] our visual aids fulfilled several important criteria including systematic development process [10,11], providing information about options, presenting probabilities, disclosing conflicts of interest, balancing the presentation of options, using plain language, and basing information on up to date scientific evidence. During presentation it was also possible to clarify values and to communicate with the presenter. There was no conflict of interest. In constrast to the requirement of decision aids our visual aids are not publicly available.

### Feasibility testing using healthy persons

After establishing the graphs as described above and previously reported [10,11], a pilot experiment was performed to test the feasibility of the visual aids. The preferences of healthy persons with regard to the treatment options were also evaluated. Thus, the visual aids of the four scenarios were presented to 213 healthy persons as a powerpoint presentation in an auditorium after a 30-minute lecture about colorectal cancer. Each participant marked his/her preferred treatment option on a form using a pencil directly after the presentation of each study. They had the option to vote for treatment A or treatment B. In addition, participants had the possibility to mark a third option, “I don't know”, if they were not able to make a decision immediately. This experiment was carried out with the support of several social clubs (Landfrauen and protestant church) in the area which organized the recruitment and transport of the volunteers to the auditorium.

### Evaluation of the visual aids by patients

After demonstrating feasibility an ethics request was filed as a precondition for the following study involving colorectal cancer patients. The study was approved by the ethics committee of the University of Ulm (no. 141/08).

After giving written informed consent, patients with colorectal cancer were questioned individually by one of the authors (J.V.) about their treatment preferences after being shown the colored print-out diagrams wrapped in transparent foil. Patients with colorectal cancer were asked to participate either during an outpatient visit at the time of follow-up 6 to 24 months after surgery (n = 52) or during inpatient treatment approximately 10 days following surgery of primary colorectal cancer (n = 50). An additional 52 healthy persons of the authors’ milieu were also questioned individually to rule out potential differences with regard to the mode of presentation (group vs. individual). Similar to the healthy persons in the auditorium, participants had the option to vote for treatment A or treatment B. They could also mark the third option, “I don't know”, if they were not able to make a decision during the individual presentation. All participants were requested to fill out a demographic questionnaire before the presentation.

## Results

### Design of the visual aids

**Figure 2:**
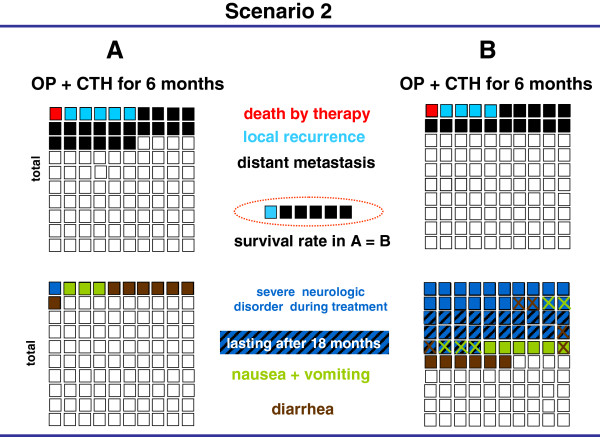
**Visual aid illustrating two adjuvant treatment scenarios for patients with UICC stages II and III colon cancer[**[14]**].** The results of an operation (OP) plus 5-FU chemotherapy (CHT) are shown as mild treatment (**A**) on the left, and the results of an OP plus 5-FU and oxaliplatin combination CHT are shown as intense treatment (**B**) on the right. The relative incidence of death, local recurrence and distant metastasis are displayed in the two upper quadrants and important undesired events in the two lower quadrants. The difference of recurrence of the two treatment options is summarized in the central dotted circle followed by a statement about overall survival benefit.

**Figure 3:**
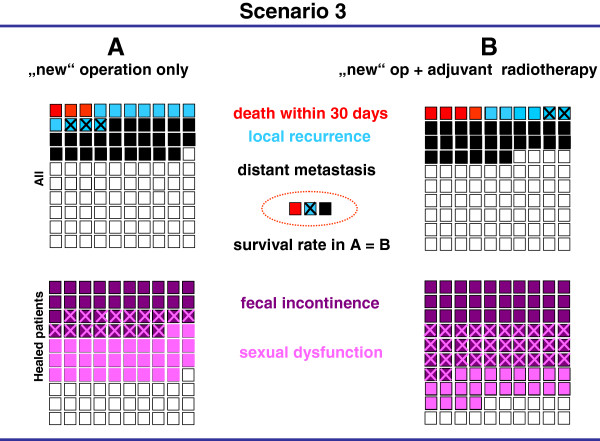
**Visual aid illustrating two treatment scenarios for patients with UICC stages I to III rectal cancer based on the published results and side effects[**[15-18]**].** The results of the “new” operation (OP) alone (total mesorectal excision technique) are shown as mild treatment (**A**) on the left, and the results of the “new” OP plus neoadjuvant short-course (5x5 Gy) radiotherapy are shown as intense treatment (**B**) on the right. The relative incidence of death, local recurrence and distant metastasis are displayed in the two upper quadrants and important undesired events in the two lower quadrants. The difference of recurrence of the two treatment options is summarized in the central dotted circle followed by a statement about an overall survival benefit.

**Figure 4:**
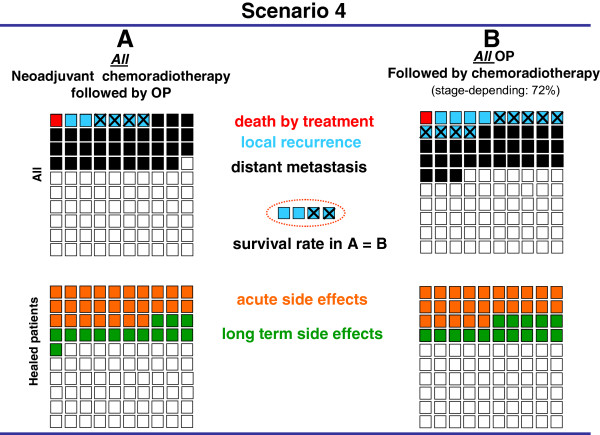
**Visual aid illustrating two equally intense treatment scenarios for patients with UICC stages I to III rectal cancer[**[19]**].** The results of neoadjuvant chemoradiotherapy followed by an operation (OP) are shown as pre-operative option (**A**) on the left, and the results of an OP followed by adjuvant chemoradiotherapy are shown as post-operative option (**B**) on the right. While all patients underwent neoadjuvant treatment in option A, only 72% of the patients underwent additional adjuvant treatment, depending on the pathological tumor stage. The relative incidence of death, local recurrence and distant metastasis are displayed in the two upper quadrants and important undesired acute and long-term side effects in the two lower quadrants. The difference of recurrence of the two treatment options is summarized in the central dotted circle followed by a statement about an overall survival benefit.

The results of four prospective, randomized controlled trials [12,14,15,19] comparing two treatment options were presented as graphic scenarios displaying outcome parameters (mortality, recurrence rates, survival) and side effects (Figures 1, 2, 3, 4). In scenarios 1 – 3, a mild therapeutic option was compared with a more intensive therapeutic option, while two equally intense options with different timing (neoadjuvant vs. adjuvant) were presented in scenario 4. The characteristics of the scenarios and related studies are summarized in Table 1, while the visual aids are shown in Figures 1, 2, 3, 4.

### Groups of participants

In a pilot experiment the prepared visual aids were presented to 213 healthy persons as a powerpoint presentation in an auditorium after a 30-minute lecture about colorectal cancer. Demonstrating the feasibility of our visual aids in a next step patients with colorectal cancer (n = 102) were questioned individually. One of the authors (J.V.) interviewed patients either during inpatient treatment following an operation (n = 50) or during an outpatient visit within the follow-up program (n = 52). The visual aids were presented as colored print-out diagrams wrapped in transparent foil. In addition, another 52 healthy persons were questioned in this individual fashion to rule out a possible influence of the mode of presentation (auditorium vs. individual). The demographics of the participating groups are summarized in Table 2.

**Table 2:** Demographic data of participating groups

		**Patients**	**Healthy Persons (individual)**	**Healthy Persons (in auditorium)**
		**n = 102**	**n = 52**	**n = 213**
**Age** (years)	20-29	24 (11%)	2 (4%)	1 (1%)
	30-39	6 (3%)	3 (6%)	2 (2%)
	40-49	26 (12%)	4 (8%)	10 (10%)
	50-59	59 (28%)	13 (25%)	17 (17%)
	60-69	51 (24%)	13 (25%)	29 (28%)
	>69	47 (22%)	17 (33%)	43 (42%)
**Gender**	female	155 (73%)	25 (48%)	41 (40%)
	male	58 (22%)	27 (52%)	61 (60%)
**Education**	secondary general school	114 (54%)	35 (67%)	65 (64%)
	intermediate school	47 (22%)	8 (15%)	17 (17%)
	grammar school	27 (13%)	2 (4%)	7 (7%)
	university	21 (10%)	7 (13%)	12 (12%)
	university + doctoral thesis	4 (2%)	0 (0%)	1 (1%)
**Relation of education to healthcare services**	no relation	154 (72%)	42 (81%)	95 (93%)
	some relation	7 (3%)	4 (8%)	3 (3%)
	relation, no treatment of patients	29 (14%)	4 (8%)	2 (2%)
	patient treatment, no physician	17 (8%)	1 (2%)	1 (1%)
	physician	6 (3%)	1 (2%)	1 (1%)
**Relation of work to healthcare services**	no relation	168 (79%)	44 (85%)	84 (82%)
	some relation	7 (3%)	3 (6%)	9 (9%)
	relation, no treatment of patients	22 (10%)	3 (6%)	6 (6%)
	patient treatment, no physician	15 (7%)	1 (2%)	2 (2%)
	working in a surgical field	1(0,5%)	1 (2%)	0 (0%)
	non-surgical field	-	0 (0%)	1 (1%)

### Decisions of the groups

The participants generated a total of 1468 preferences when shown the 4 scenarios – 408 patient preferences and 1060 preferences of healthy persons. The results for the three groups are summarized in Table 3. There were no differences in the decisions between the in- and outpatients (data not shown).

**Table 3:** Results of test person’s decisions

	**Patients**			**Healthy Persons (individually asked)**			**Healthy Persons (in auditorium)**		
	**n = 102**			**n = 52**			**n = 213**		
	**Treatment option**			**Treatment option**			**Treatment option**		
**Scenario**	**Mild**	**Intense**	**No decision**	**Mild**	**Intense**	**No decision**	**Mild**	**Intense**	**No decision**
**1**[12,13]	23 (23%)	79 (77%)	0 (0%)	15 (29%)	37 (71%)	0 (0%)	102 (48%)	105 (49%)	6 (3%)
**2**[14]	81 (80%)	19 (18%)	2 (2%)	46 (88%)	6 (12%)	0 (0%)	192 (90%)	9 (4%)	12 (6%)
**3**[15-18]	84 (82%)	12 (12%)	6 (6%)	42 (81%)	4 (8%)	6 (11%)	166 (78%)	21 (10%)	26 (12%)
**Scenario**	**Pre-OP**	**Post-OP**	**No decision**	**Pre-OP**	**Post-OP**	**No decision**	**Pre-OP**	**Post-OP**	**No decision**
**4**[19]	42 (41%)	57 (56%)	3 (3%)	23 (44%)	24 (46%)	5 (10%)	60 (28%)	138 (65%)	15 (7%)

The first scenario (Figure 1) compared an operation alone with an operation and adjuvant mono-chemotherapy in colon cancer [12]. The results were characterized by a 5% survival advantage in the second group. Furthermore, an obviously lower rate for local recurrence and distant metastasis could be shown. Side effects were only documented for the chemotherapy group. The majority of patients and healthy persons questioned individually chose the intensive treatment (77% and 71%, respectively). Only half of the healthy persons from the auditorium group chose this option (49%).

The second scenario (Figure 2) displayed two adjuvant treatment options for colon cancer, comparing 5-FU mono-chemotherapy with 5-FU and oxaliplatin double-chemotherapy [14]. Despite better control of tumor recurrence by the intensive option, survival rates in the overall group (UICC II and III) were not affected according to the data published in 2004. All groups showed a strong preference for the mild treatment option, irrespective of the mode of presentation (range 81% - 90%, Table 3).

In the third scenario (Figure 3), the operation alone involving a total mesorectal excision (TME) was compared with short-course radiotherapy (5x5 Gy) followed by TME in rectal cancer [15]. The intensive option showed better local control and a somewhat lower frequency of distant metastases, but no effect on survival. The intensive option was associated with more side effects. Similar to scenario 2, all groups preferred the mild treatment option, regardless of the form of presentation (range 78% - 82%, Table 3).

In the fourth scenario (Figure 4), two equally intense treatment options comparing neoadjuvant chemoradiotherapy with adjuvant chemoradiotherapy in rectal cancer were presented [19]. The first option included all patients in the neoadjuvant treatment, whereas the second option included only 70% of the patients - depending on the tumor stage – who received adjuvant chemoradiotherapy. The advantage of the neoadjuvant option was the lower rate of local recurrence and distant metastasis (Figure 4). The groups of patients and healthy persons questioned showed no preference, choosing the adjuvant treatment option in 65% to 46%, respectively. There was no difference between the preference of any group.

## Discussion

Three main forms of patient-physician relationships have been described [2]: First, the era of parternalism - doctor served as the guardian; second, the era of informed consent; and third, the era of shared-decision-making. Today many patients want to be well informed and demand to take an active role in the planning of their treatment reflecting a shift in the patient’s role in decision-making from the passive bystander to an active participant [2,3,6]. However, patients’ desire for autonomy is impaired by a lack of disease-specific knowledge and by the growing complexity of treatment options which have recently become available, especially in cancer therapy. Guidelines have been developed by expert consensus to give recommendations for a therapy or combination of therapies according to the disease stage to support medical professionals in their recommendations. However, since guidelines are standardized, they may not consider the patient’s individual preferences and thoughts about life planning. To be able to participate in the individual decision-making process it is essential to provide information for the patients based on current scientific evidence that is both easy to understand and detailed. No studies about the use of decision aids in neoadjuvant or adjuvant treatment of potentially curative colorectal cancer have been published to date [8]. Therefore, in analogy to decision aids we designed visual aids for neoadjuvant and adjuvant treatment of colorectal cancer depicting various therapy options. The design of our visual graphs basically followed the basic recommendations for decision aids standards except public availability [20,24]. Our aim was to evaluate the treatment preferences of patients for these multimodal treatment options using visual aids.

To test the feasibility of the visual aids the four designed graphs were presented to 213 healthy persons in an auditorium after a lecture about colorectal cancer. These preliminary results demonstrated that our visual aids were feasible, because most participants could make decisions immediately. Evaluation of these results further revealed that healthy persons generally preferred the milder treatment options. These options were often not recommended by our German guideline [25]. In order to rule out that healthy persons decide different from patients suffering from colorectal cancer facing a potentially lethal disease the second part of our study was carried out. Patients were asked individually for their preferences. We again asked healthy persons in an individual manner to rule out effects with regard to the mode of presentation.

Our results demonstrated that most patients and healthy persons can make a decision after being shown the visual aids. As expected the majority of patients (77%) and healthy persons (71%) chose the intensive treatment option in scenario 1 demonstrating a survival benefit when asked individually. We do not know why only 49% of healthy persons questioned in the auditorium chose the option accompanied with the survival benefit. It is possible therefore that the presenter and mode of presentation may have influenced the decision making to some extent, one possible limitation of our study. It is also possible that the lecture about colorectal cancer might have affected their opinion.

In contrast, the evaluation of the treatment choices without effect on overall survival revealed that the majority of the patients and healthy persons preferred the mild treatment options (78%-90%) independent of the mode of presentation (scenarios 2 and 3). When looking at the graphs of the visual aids these preferences seem not really surprising. However, they clearly contradict the opinion of specialists justifying intensive treatment by a sole reduction of recurrence rates and the present German guideline recommendations [25]. Our results suggest that participants - patients and healthy persons - placed more emphasis on quality-of-life aspects than on recurrence rates in decisions without difference in survival. This was not surprising to us and confirmed our previous results in healthy persons [10,11,26].

The fourth scenario depicted two equally intense options comparing different modes of application (neoadjuvant vs. adjuvant) in rectal cancer. The advantage of the neoadjuvant option is the significantly lower rate of local recurrence. This option is therefore recommended presently by the treatment guidelines and most physicians [25]. However, patients and healthy persons did not show a preference for the neoadjuvant therapy (28%-41%). Instead, no difference in the treatment preference was observed between the neoadjuvant and adjuvant options presented. This is probably related to the lack of a survival benefit and the possibility to avoid chemoradiotherapy at all when choosing the other option (adjuvant). While all patients received neoadjuvant chemoradiotherapy, only 72% of the patients received adjuvant chemoradiotherapy. The remaining 28% of the patients had tumors of lower stage that did not require multimodal treatment after final pathological examination [19].

In summary, our results showed: first, that patients and healthy persons can make decisions about presented multimodal treatment options using visual aids; second, that the decisions of patients and healthy persons are equal and independent of the mode of presentation when comparing mild versus intensive treatment without survival benefit; third, in decisions with survival benefit, patients and healthy persons prefer the beneficial option, but this may depend on the mode of presentation; and fourth, there is no preference for “equally” intense options without survival benefit.

## Conclusions

We conclude from our results and experience gained during the project that patients’ decisions are primarily influenced by quality-of-live aspects and overall survival, often contrasting treatment recommendations made by specialists and national guidelines. Visual aids may be reasonable tools in the management of colorectal cancer patients in the future. They may be helpful to supply patients with additional information about the results and side effects of various disease-specific treatment options thereby increasing patients’ knowledge about the disease and thus gaining more confidence in the treatment and its consequences.

## Competing interests

The authors declare that they have no competing interest.

## Authors’ contributions

KM, PF - conception and design; VJ, WC, HBD, PF, KM - provision of study materials or patients; VJ, WC, KM - collection and assembly of data; HS, HBD, PF, KM - data analysis and interpretation; HS, PF, KM - manuscript writing. All authors read and approved the final manuscript.

## Pre-publication history

The pre-publication history for this paper can be accessed here:

http://www.biomedcentral.com/1472-6947/12/118/prepub
